# Integrated Time-Series Biochemical, Transcriptomic and Metabolomic Analyses Reveal Key Pathways and Concentration-Dependent Transitions in Chironomid Larvae (*Propsilocerus akamusi*) Under Chlorantraniliprole Stress

**DOI:** 10.3390/ani16172779

**Published:** 2026-09-03

**Authors:** Jiani Li, Wei Chen, Jian Mao, Runze Li, Chuncai Yan, Zeyang Sun

**Affiliations:** College of Life Sciences, Tianjin Normal University, Tianjin 300387, China; ljn18602604560@163.com (J.L.); 15620272533@163.com (W.C.); 18522445361@163.com (J.M.); lrz17627870325@126.com (R.L.); skyycc@tjnu.edu.cn (C.Y.)

**Keywords:** chironomid larvae, chlorantraniliprole stress, protein homeostasis, Hsp70

## Abstract

Chironomids are abundant aquatic macroinvertebrates with known pollutant tolerance, but the molecular effects of chlorantraniliprole (CAP) remain unclear. In this study, we exposed *Propsilocerus akamusi* larvae to LC_10_ and LC_50_ of CAP, sampled at 48 h and 144 h, and integrated biochemical assays, metabolomics, and transcriptomics to profile stress responses. Our results showed that CAP elevated antioxidant enzymes and protein carbonyls, indicating severe oxidation, especially at LC_50_. At 48 h, LC_10_ activated JNK-mediated antioxidative and drug metabolism pathways, whereas LC_50_ altered glycolysis, cofactor biosynthesis, and fatty acid metabolism. At 144 h, LC_10_ enriched proteasome and oxidative phosphorylation pathways; chaperones *PaHsp70* and *PaHsp90* were upregulated by 4.14- and 2.08-fold, respectively, and were negatively correlated with lipid and carbohydrate metabolites, indicating a metabolic shift toward protein repair. In contrast, LC_50_ CAP triggered protein processing in the endoplasmic reticulum (ER) and TCA cycle dysregulation, with upregulation of PaCalreticulin linked to energy crisis markers, reflecting severe energy collapse. After knockdown of *PaHsp70*, the survival rates of chironomid larvae exposed to LC_10_ and LC_50_ CAP significantly decreased from 80.8% to 65.8% and from 43.3% to 28.3%, respectively. Collectively, our results revealed that low CAP concentrations triggered early detoxification followed by proteasome- and Hsp-mediated strategies to sustain protein homeostasis whereas high concentrations directly disrupted core metabolism and ultimately caused energy collapse and lethal ER stress in chironomid larvae. These findings provide mechanistic insights into the chronic effects of CAP on aquatic insects.

## Simple Summary

Chironomids are among the most abundant and diverse freshwater insects, and are valued as pollution indicators and as key components of aquatic food webs. The widespread use of pesticides in agriculture has raised serious concerns about their effects on these aquatic organisms. To address this, we studied how a common insecticide, chlorantraniliprole (CAP), at environmentally relevant concentrations, affected chironomid larvae. Our findings showed that low doses of CAP triggered protective mechanisms in the larvae, such as detoxification and protein repair systems, to cope with stress. However, higher doses overwhelmed these defenses, disrupting energy production and causing severe cellular stress that ultimately led to death. We identified a key protein, *PaHsp70*, that was essential for larval survival under insecticide exposure. When its expression was significantly reduced, the larvae became more vulnerable. These findings reveal the adaptive strategies of chironomids to cope with pesticide stress from the environment, and highlight the urgent need to protect these animals and their freshwater habitats from agricultural pollution.

## 1. Introduction

Chlorantraniliprole (CAP) belongs to the diamide insecticide family. It is a novel insecticide that targets the ryanodine receptor and effectively controls pest populations, including those resistant to other insecticides [1]. Upon activation of the ryanodine receptor, CAP causes sustained opening of calcium channels in the smooth and striated muscles of insects, which leads to muscle paralysis, relaxation, rigidity, and ultimately death [2,3]. CAP is resistant to photolysis and hydrolysis in water, which allows it to persist and contaminate water sources. It has been reported that the majority of initially applied CAP can persist in flooded paddy fields for up to 20 days, with surface water concentrations ranging from 0.04 to 950 μg/L [4]. Consequently, the potential effects of CAP on non-target aquatic organisms have garnered considerable attention. For instance, exposure to CAP has been shown to affect the oxidative system in tadpoles of *Boana faber* [5]. In grass carp *Ctenopharyngodon idella*, CAP triggers mitochondrial dysfunction by altering mtROS and mitochondrial dynamics [6]. Acute challenge by CAP could cause immunosuppressive toxicity in silver catfish *Rhamdia quelen* facing a bacterial infection [7]. CAP treatment induced detoxification inhibition in the liver of crucian carp *Carassius carassius* and disrupted the gut flora composition [8].

Chironomids belong to the order Diptera and are dominant benthic macroinvertebrates in freshwater ecosystems, constituting a key dietary component for amphibians, fish, and avian predators [9]. Their life cycle comprises four stages, including egg, larva, pupa, and adult. The larval stage is the longest, during which larvae typically feed on organic detritus, algae, and bacteria at the bottom of water bodies [10]. By doing so, they can improve water quality, accelerate organic matter mineralization in the material cycle of water bodies, and eliminate organic pollution. Research has shown that chironomid larvae typically build tubular structures from salivary silk and soft benthic particles [11]. Despite inhabiting these tubes, the larvae extend their heads or anterior parts above the sediment surface to feed or uptake oxygen, thereby becoming directly exposed to environmental stressors. Both the US Environmental Protection Agency (USEPA) and the Organisation for Economic Co-operation and Development (OECD) have designated typical Chironomidae species as a recommended test species for assessing environmental pollutants in freshwater invertebrates owing to their greater sensitivity to aquatic contaminants than terrestrial and other invertebrate groups. Consequently, extensive toxicological research has been conducted on chironomid larvae to systematically characterize their responses. For instance, exposure to the common cyanobacterium Microcystis suppresses burrowing activity and life history traits of *Chironomus riparius* [12]. The deleterious effects of 2,4-dichlorophenoxyacetic acid disrupted survival, emergence, and fecundity in *C. sancticaroli* [13], as well as the endocrine system and DNA repair processes in *C. riparius* [14]. Through evolution, various chironomids inhabiting different niches have developed specific strategies to achieve high tolerance to environmental stressors, such as heavy metals, high temperatures, dehydration, and pesticides [15,16,17,18]. As in vertebrates, chironomids rely on antioxidant defenses and detoxification pathways to cope with pollutant stress. Antioxidant enzymes quench excess ROS, whereas detoxifying enzymes promote the excretion of xenobiotics by increasing their solubility [19,20,21]. In addition, cuticular proteins involved in structural enhancement and metallothioneins play key roles in heavy metal tolerance [18,22]. Furthermore, synergistic interactions with gut microbiota have recently been elucidated as an additional protective mechanism. Symbionts can alleviate the toxicity of pesticides, heavy metals, and cyanobacterial toxins by promoting xenobiotic bioremediation, enhancing host detoxification enzyme activity, and maintaining intestinal barrier integrity [17,23,24,25]. Even though some previous investigations have elucidated the effects of CAP on enzyme activities of chironomids [26,27], the molecular-level implications of exposure to environmentally relevant concentrations and semi-lethal doses of CAP in chironomid larvae remain poorly understood. Elucidating these implications would facilitate a better understanding of the toxic mechanisms of CAP and the potential resistance mechanisms of the host.

As a crucial approach in environmental research, multi-omics is indispensable for identifying molecular alterations induced by pollutant exposure, and within this framework, Weighted Gene Co-expression Network Analysis (WGCNA) is commonly used to enable deep-level gene mining and to provide a basis for exploring gene functions under stress conditions. For example, a small heat shock protein, alpha-crystallin A, from *Arma chinensis* was highlighted as a central hub gene in a temperature-tolerant module of the co-expression network [28]. In the invasive fruit fly *Bactrocera dorsalis*, WGCNA uncovered important modules associated with acute cold tolerance and identified a chitin-binding gene as a critical network component [29]. In addition, combined transcriptome and metabolome analysis has been extensively applied in insects to reveal stress response characteristics at both the gene expression and metabolite levels. This integrative strategy enables the discovery of key regulatory pathways that are otherwise obscured when using a single omics approach, providing a systems-level understanding of insect response to stressors [30]. However, the molecular network underlying CAP stress response in chironomid larvae has not been characterized. WGCNA offers an unbiased approach to identify co-expressed gene modules and hub regulators that orchestrate the temporal transition from adaptation to toxicity. Here, CAP was used as an environmental stressor on *Propsilocerus akamusi* (Tokunaga) larvae, which is a dominant and ubiquitous chironomid species in East Asia. Its larvae are large and morphologically unique, and are considered indicator organisms for detecting water contamination and potential toxicity. While our recent work focused on CAP-induced gut microbiota dysbiosis in this species [17], the present work integrates multi-omics with WGCNA and provides functional validation via RNAi, which moves beyond correlative observations to establish causal mechanistic links. We aimed to characterize both the adaptive mechanisms that mitigate CAP stress and the toxicological outcomes when these defenses are overwhelmed.

## 2. Methods

### 2.1. Experimental Organisms, Rearing Conditions and Test Design

*P. akamusi* larvae were collected from natural freshwater environments and identified via morphological characteristics. The larvae were acclimated in an artificial climate chamber at 21 ± 1 °C under a 16 h light: 8 h dark photoperiod. Dechlorinated tap water with sufficient aeration was used for cultivation, and larvae were fed daily with ground tropical fish Tetramin^®^ (Tetra Werke, Melle, Germany) to ensure normal feeding and growth. Prior to exposure, healthy fourth-instar larvae of uniform body size (14–17 mm) and good vitality were selected as experimental subjects.

A CAP standard with a purity higher than 95% (CAS: 500008-45-7, Kaiwei Chemical, Shanghai, China) was used in this study. Exposure solutions were prepared with 0.1% acetone as the cosolvent. As determined from our previous studies, the 144-h LC_10_ and LC_50_ values of CAP against *P. akamusi* larvae were 5 and 50 µg/L, respectively [17] (Table S1). Therefore, the control group, low-concentration treatment group (LC_10_), and high-concentration treatment group (LC_50_) were established. A final acetone concentration of 0.1% was maintained in all groups to eliminate solvent effects. Approximately two-thirds of the fresh exposure solution was replaced every 24 h to keep the concentration stable. Larval samples were collected with sterile forceps and rinsed quickly with sterile PBS buffer to remove residual chemicals and impurities. For biochemical assays, larvae were sampled at 24, 48, 96, and 144 h to monitor temporal physiological dynamics. For transcriptomic analyses, larvae were sampled at 48 h and 144 h to represent acute and subchronic phases of stress response, respectively.

### 2.2. Determination of Oxidative Biomarkers

Biochemical assays were performed on larvae from each treatment group at four time points, including 24 h, 48 h, 96 h and 144 h after CAP exposure. Three independent biological replicates were arranged. For every single replicate, five chironomid larvae were collected and fully homogenized in pre-cooled sterile PBS. All homogenized samples were centrifuged at 4000 rpm for 10 min under 4 °C constant temperature. After centrifugation, the supernatant liquid was carefully harvested and reserved for subsequent biochemical detection. The enzymatic activities of glutathione peroxidase (GSH-Px) and peroxidase (POD), as well as the content of protein carbonyl, were determined using commercially available assay kits purchased from Nanjing Jiancheng Bioengineering Institute. All quantitative measurements in this experiment were completed by spectrophotometric analysis to guarantee stable and unified detection conditions among all samples.

### 2.3. RNA Extraction, Transcriptome Sequencing and Analysis

A total of four larvae from each group were homogenized into powder and each group contained six replicates. TRIzol reagent and chloroform were used for total RNA extraction, with phase separation achieved by centrifugation. The RNA was then precipitated with isopropanol, washed with 75% ethanol, and finally dissolved in TE buffer. To confirm RNA integrity and concentration, we performed 1% agarose gel electrophoresis and used an Agilent 5300 Bioanalyzer (Agilent Technologies, Santa Clara, CA, USA). All samples had RNA concentrations greater than 500 ng/μL. RNA processing and sequencing were conducted by Shanghai Majorbio Bio-pharm Biotechnology Co., Ltd. (Shanghai, China). A strand-specific RNA-seq library was prepared from 1 μg of total RNA employing the Illumina^®^ Stranded mRNA Prep Ligation Kit (Illumina Inc., San Diego, CA, USA). The process started with poly(A)-based mRNA capture using oligo(dT) magnetic beads, followed by fragmenting the RNA. First-strand cDNA was synthesized with random hexamer primers, and second-strand synthesis was subsequently carried out. After end repair, adenylation, and adapter ligation on the double-stranded cDNA, size selection was performed using magnetic beads. The selected fragments were then amplified over 10 to 15 PCR cycles. The final library was quantified via a Qubit 4.0 (Thermo Fisher Scientific, Waltham, MA, USA) and submitted for PE150 sequencing on the Illumina NovaSeq X Plus system (Illumina Inc., San Diego, CA, USA).

Raw sequencing data were subjected to quality evaluation and filtering to remove low-quality reads, adapter sequences and reads with ambiguous bases, yielding high-quality clean reads. Clean reads were mapped to the reference genome of *P. akamusi* (Bioproject accession: PRJNA698390), and gene expression levels were calculated using FPKM as the quantitative index to construct a gene expression matrix. Data normalization, outlier elimination and missing value imputation were performed in R (version 4.5.0) to ensure reliability. The Benjamini–Hochberg (BH) method was applied to correct raw *p* values and control the false discovery rate (FDR). Differentially expressed genes (DEGs) were identified against the control group with thresholds of |log_2_FC| > 1 and FDR < 0.05. Volcano plots were constructed in R to visualize the distribution of up- and down-regulated genes, and the results were used for functional annotation and enrichment analysis. To further reveal the changes in key metabolic pathways in larvae under different concentrations of CAP stress, KEGG pathway enrichment analysis was performed for DEGs in low- and high-concentration groups at each time point. DEG sequences were aligned to the KEGG database for pathway annotation and enrichment statistics. Pathways with *p* < 0.05 were considered significantly enriched, which helped identify markedly disturbed metabolic and signaling pathways and provided functional support for subsequent analysis.

### 2.4. WGCNA

WGCNA clusters genes with highly consistent expression trends into co-expression modules based on expression similarity across multiple samples. Based on FPKM data from different treatment groups and time points, a co-expression network was constructed using the WGCNA package (version 1.74) in R (version 4.5.0). All samples were subjected to hierarchical clustering to remove outliers. A soft threshold β = 18 was determined to satisfy the scale-free network feature. An adjacency matrix was constructed and transformed into a topological overlap matrix (TOM). Genes were clustered hierarchically according to correlation strength, and modules were detected using the dynamic hybrid cutting algorithm. The minimum module size was set to 40, and modules with highly similar expression patterns were merged with a merge cutoff of 0.2. Module eigengenes were calculated and correlated with phenotypic indicators including exposure concentration, duration and metabolite contents to identify key stress-related modules. Gene significance (GS) and module membership (MM) were calculated, and genes satisfying |MM| > 0.5, |GS| > 0.2 and *p* < 0.05 were defined as hub genes. The yellow, brown and blue modules, strongly correlated with CAP exposure, initially contained 249, 474 and 626 genes, respectively. A total of 30 core hub genes in key modules were screened according to weighted connectivity, and the core gene network was visualized using Cytoscape 3.10.4.

### 2.5. Metabolite Extraction and Metabolomics Analysis

Non-targeted metabolomic profiling was conducted on pooled larval specimens after 144 h exposure. Six replicates were established per experimental group, each replicate consisting of 12 larvae chosen at random. From each replicate, 100 mg of tissue was added to 500 μL of a methanol-water mixture (4:1, *v*/*v*) for metabolite extraction. The sample was subsequently ground at −10 °C and 50 Hz for 6 min using a Wonbio-96c frozen tissue grinder (Shanghai Wanbo Biotechnology Co., Ltd., Shanghai, China), then subjected to low-temperature ultrasonic extraction (5 °C, 40 kHz) for 30 min. After a 30-min incubation at −20 °C, centrifugation was performed at 13,000× *g* for 15 min. The clear supernatant was collected and analyzed by LC-MS/MS on a UHPLC-Q Exactive HF-X system fitted with an ACQUITY HSS T3 column (100 mm × 2.1 mm i.d., 1.8 μm; Waters, Milford, MA, USA). The analysis was carried out at Majorbio Bio-Pharm Technology Co., Ltd. (Shanghai, China). Differential metabolites (DEMs) were defined as those having a VIP > 1, |FC| > 1.5, and *p* < 0.05. Putative annotation of these metabolites was achieved by cross-referencing with the Human Metabolome Database, LlPlD MAPS Structure Database, and KEGG database.

### 2.6. Gene-Metabolite Correlation Analysis and Interaction Network Construction

To systematically analyze the coordinated changes between gene expression and metabolite accumulation, correlation analysis was performed between DEGs and DEMs after 144 h exposure to LC_10_ and LC_50_ CAP. The top 10 DEGs and top 5 DEMs were screened by ascending *p* value for correlation analysis. Expression and content data were merged into an integrated matrix; missing values were filled with 0, and data were standardized using the log_2_ (x + 1) transformation. The Pearson correlation coefficient was used to evaluate the correlation strength between genes and metabolites. Significant gene–metabolite pairs were organized into node and relationship files. These files were then imported into Cytoscape 3.10.4 to construct gene-metabolite interaction networks for LC_10_ and LC_50_ CAP groups at 144 h, so as to directly compare the differences in molecular regulatory networks under different CAP stress levels.

### 2.7. Reverse Transcription Quantitative Polymerase Chain Reaction (RT-qPCR) Validation for Candidate Differentially Expressed Genes

Reverse transcription was performed in a 10 μL reaction mixture containing 2 μL of RT Master Mix, 5 μL of RNA extract, and 3 μL of ddH_2_O. For RT-qPCR, a 20 µL reaction mixture was set up in triplicate. The mixture consisted of 10 µL of SYBR Green Master mix (Takara, Shanghai, China), 0.4 µL of forward primer (10 µM), 0.4 µL of reverse primer (10 µM), 3 µL of cDNA template, and 6.2 µL of ddH_2_O. The primers used for the target genes are listed in Table 1. Relative expression levels of the genes were determined by normalizing the Ct value of each sample to that of the housekeeping gene β-actin, using the 2^−∆∆Ct^ method.

### 2.8. The Structural Analysis of PaHsp70

Since no experimental HSP70 crystal structure of *Propsilocerus akamusi* exists, homologous HSP70 amino acid sequences were retrieved from UniProt (web-based service, https://www.uniprot.org/, accessed on 20 August 2026). Conserved *N*-terminal ATPase and C-terminal substrate-binding domains were annotated via InterPro (web-based service, https://www.ebi.ac.uk/interpro/, accessed on 20 August 2026). The AlphaFold Hsp70 model AF_AFP09446F1 was visualized to observe domain spatial distribution.

### 2.9. RNA Interference-Mediated Knockdown of the PaHsp70 Gene and Its Functional Analysis

To generate double-stranded RNA (dsRNA) for gene silencing, a cDNA fragment of the *PaHsp70* gene (encoding the heat shock protein 70 molecular chaperone from *P. akamusi*), and a cDNA fragment of the target gene were amplified using primers containing the T7 promoter sequence (TAATACGACTCACTATAGGG) at both ends. The PCR product was purified with a commercial kit (Sangon Biotech, Shanghai, China). The dsRNA was then synthesized using the T7 RNAi Transcription Kit (Vazyme, Nanjing, China) in a 20 μL reaction containing 2 μL of 10× Transcription Buffer, 8 μL of NTP Mix, 2 μL of T7 Enzyme Mix, 2 μL of dsRNA template, and 6 μL of RNase-free ddH_2_O. After incubation at 37 °C for 2 h, the transcription product was digested with DNase I and RNase T1 (10 U/μL) for 30 min at 37 °C. The resulting dsRNA yield was over 20 μg and verified by 1% agarose gel electrophoresis. Chironomid larvae were exposed to a final dsRNA concentration of 1 μg/μL in two treatment groups for 144 h, namely *dsPaHsp70* combined with 5 μg/L CAP and *dsPaHsp70* combined with 50 μg/L CAP. The delivery of dsEGFP was used as the control. Each group consisted of three replicate wells (6-well plates) with 10 larvae per well. Air was supplied every 8 h using a pipette, and the exposure solution was replaced every 12 h to maintain stable dsRNA and CAP concentrations.

### 2.10. Data Deposition and Statistical Analysis

The raw transcriptome sequence reads could be archived in the NCBI SRA database under the accession numbers PRJNA1460572 and PRJNA1348783. The metabolome raw data has been deposited in the Metabolomics Workbench repository under the study ID ST004511. One-way ANOVA was applied to determine significant differences in oxidative markers across experimental groups. Welch’s t test was employed to measure the gene expression level and survival rate. All results are reported as mean ± standard error of the mean (SEM). A *p* value of less than 0.05 (*), 0.01 (**) and 0.001 (***) was considered statistically significant.

## 3. Results

### 3.1. The Effects of CAP Exposure on Oxidative Biomarkers

**Figure 1:**
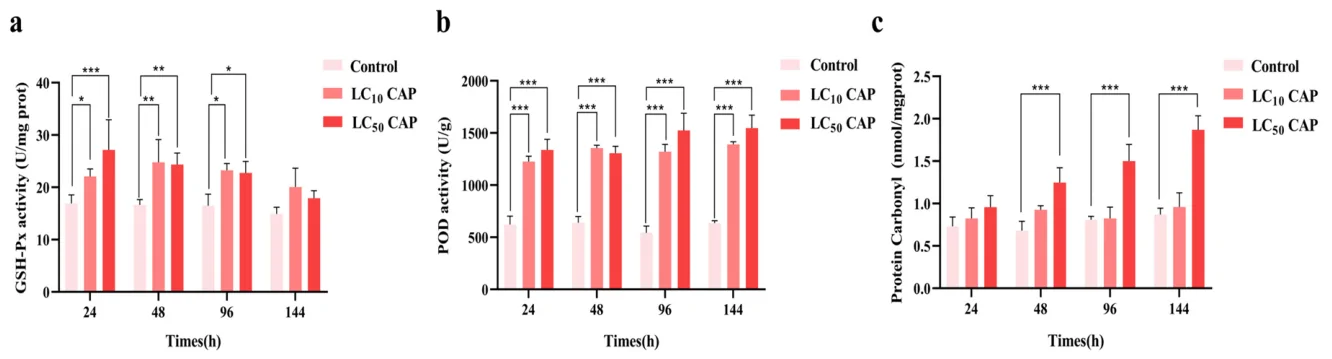
Determination of oxidative stress markers in chironomid larvae under CAP stress. (**a**) The enzyme activity of GSH-Px. (**b**) The enzyme activity of POD. (**c**) Protein carbonyl levels. Asterisks indicate differences between comparisons (*, *p* < 0.05, **, *p* < 0.01; ***, *p* < 0.001).

Exposure to CAP at both LC_10_ and LC_50_ concentrations significantly increased GSH-Px activity from 24 h to 96 h. However, while the LC_50_ group showed a statistically significant elevation, the enzyme activity exhibited a decreasing trend over this period (Figure 1a). From 24 h to 144 h of exposure, both LC_10_ and LC_50_ concentrations of CAP significantly increased POD activity. Notably, the LC_50_ concentration induced a greater enhancement in POD activity compared to the LC_10_ group (Figure 1b). From 48 to 144 h, LC_50_ CAP, instead of the LC_10_ dosage, significantly raised larval protein carbonyl levels, reflecting severe protein oxidation due to high-level pesticide invasion (Figure 1c).

### 3.2. Transcriptome Profiling and Enrichment Analysis of DEGs

In this study, three concentrations were used to treat larvae for 48 and 144 h, with three replicates per treatment group. Transcriptome analysis was performed on 18 mRNA libraries from CAP-treated and control samples using the Illumina sequencing platform. After removing adapter sequences and low-quality reads, a total of 39,149,490 to 50,256,284 clean reads were obtained. The transcriptome data showed high accuracy, with the proportion of high-quality bases achieving a Q30 score exceeding 96.3% in all samples. Moreover, more than 96.2% of the clean reads were successfully mapped to the reference genome of *P. akamusi* (Table S2).

**Figure 2:**
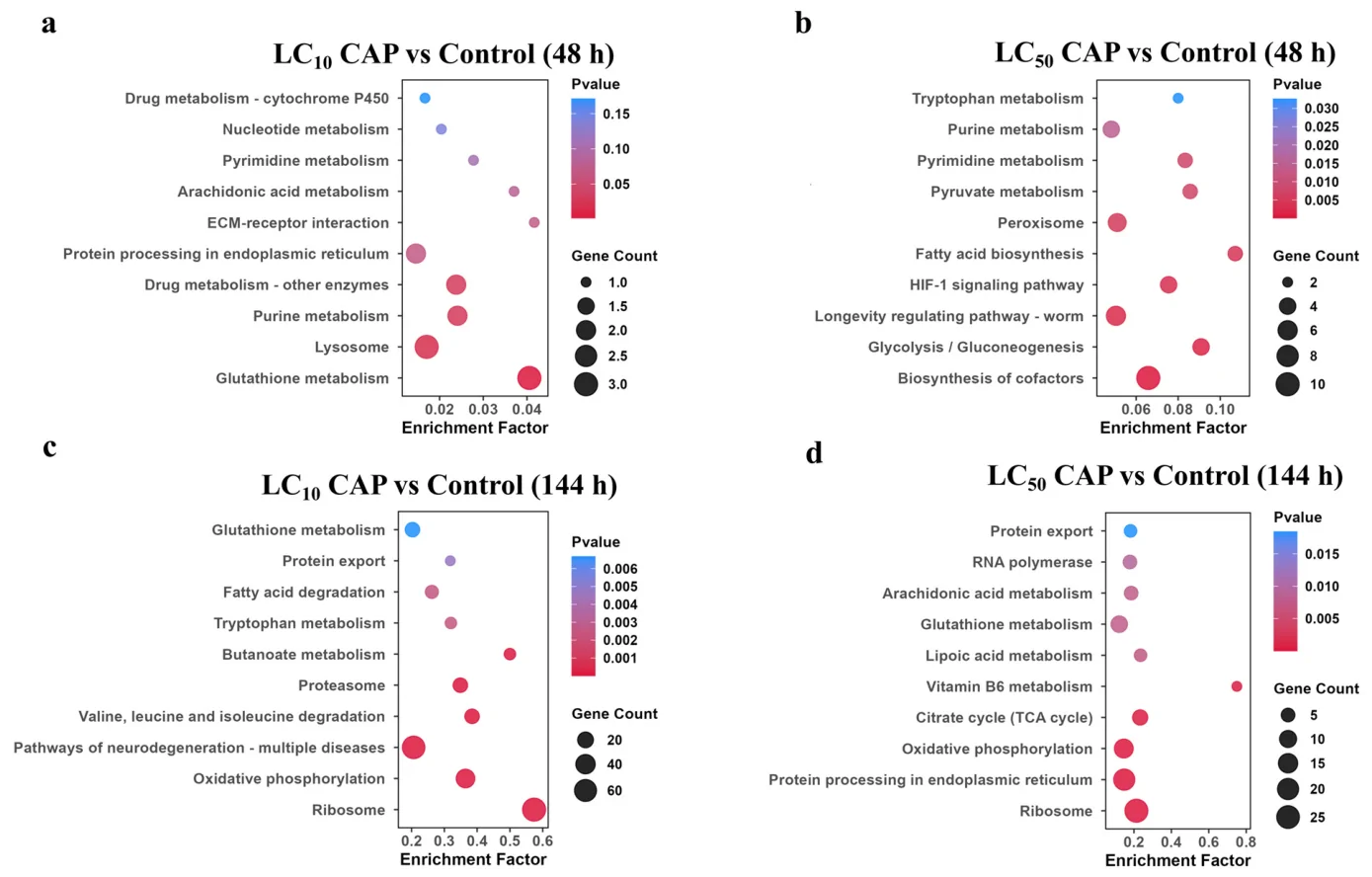
Transcriptome analysis of enriched KEGG pathways in CAP-challenged chironomid larvae. (**a**) LC_10_, 48 h vs. control. (**b**) LC_50_, 48 h vs. control. (**c**) LC_10_, 144 h vs. control. (**d**) LC_50_, 144 h vs. control.

Following treatment with LC_10_ CAP at 48 h, a total of 54 genes were identified as DEGs compared with the control group, among which 26 transcripts were upregulated and 28 were significantly downregulated. These DEGs were primarily enriched in glutathione metabolism, lysosome, purine metabolism, and drug metabolism pathways. LC_50_ CAP at the same time point yielded 289 DEGs, which were mainly assigned to biosynthesis of cofactors, glycolysis, peroxisomes, and fatty acid biosynthesis pathways. At 144 h, LC_10_ CAP induced 699 DEGs with 193 upregulated and 506 downregulated genes. After analysis, these DEGs were grouped into ribosome, oxidative phosphorylation and proteasome pathways. When the CAP dosage increased to LC_50_, 539 DEGs were identified with enrichment in ribosomes, protein processing in the endoplasmic reticulum, oxidative phosphorylation, TCA cycle and glutathione metabolism (Table 2, Figure 2).

### 3.3. Co-Expression Network Analysis

**Figure 3:**
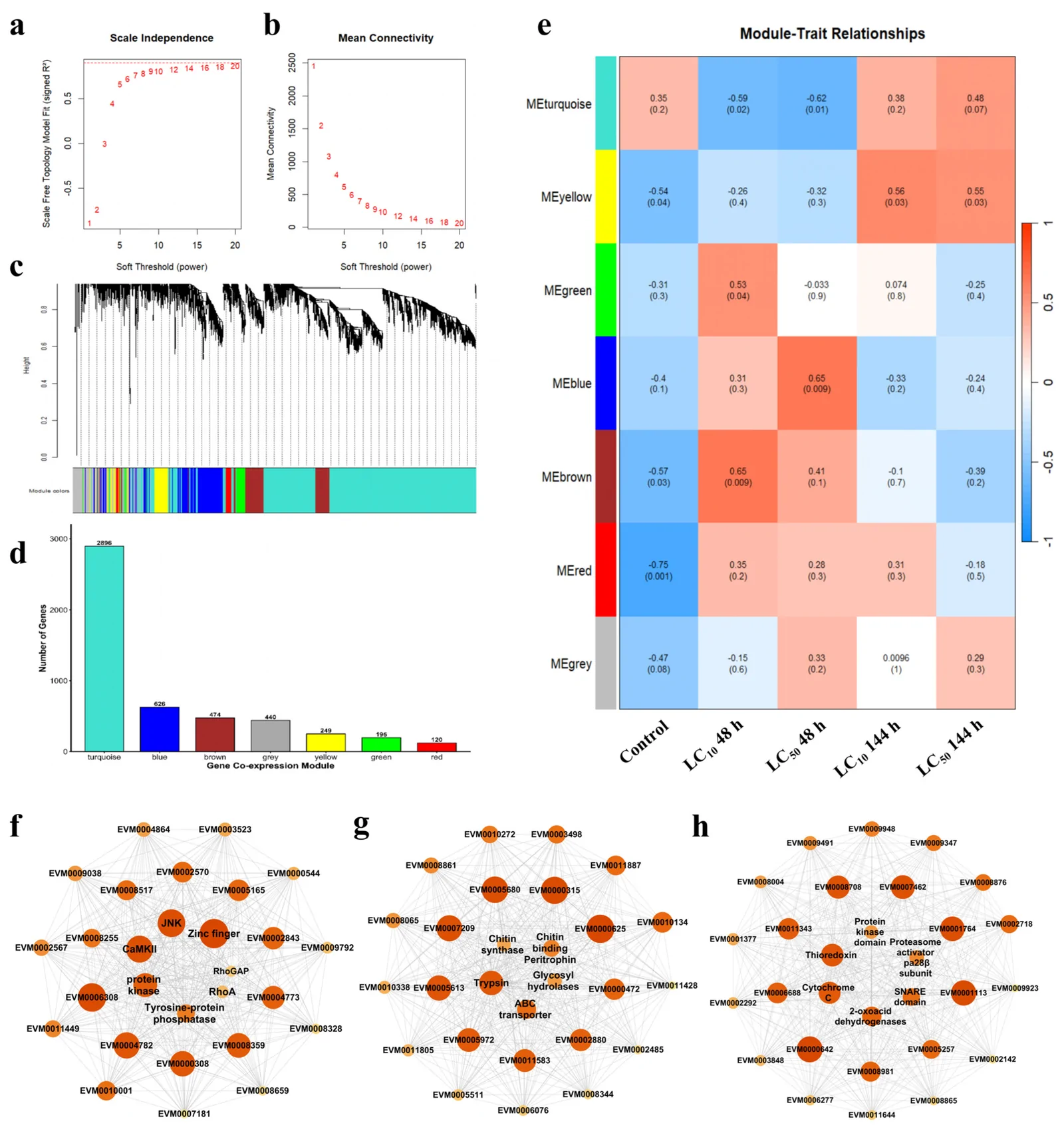
WGCNA of the genes among control and CAP treatment groups. (**a**) Scale independence analysis. (**b**) Mean connectivity analysis. (**c**) Hierarchical cluster tree showing the co-expression modules identified by WGCNA. The major branches, labeled with different colors, constitute 7 modules. (**d**) Number of genes in each module. (**e**) Correlation analysis between modules and treatment via WGCNA. The color and value in each cell represent the correlation coefficient between the modules and the treatment. (**f**) Hub genes identified in the brown module. (**g**) Hub genes identified in the blue module. (**h**) Hub genes identified in the yellow module. Node size and color both represent connectivity scores; larger and darker nodes indicate higher connectivity and greater centrality.

Based on the median absolute deviation, the top 5000 most highly expressed genes were screened to construct a weighted gene co-expression network. The soft threshold power was determined as 18 to satisfy the scale-free network property, and a total of seven gene co-expression modules were ultimately identified (Figure 3a–c). The turquoise module contained the largest number of genes, with 2896, whereas the red module contained the smallest, with only 120 (Figure 3d). Module-trait correlation analysis revealed that different modules exhibited significant time-specific responses to CAP stress. Notably, the yellow module showed significant positive correlations with both LC_10_ and LC_50_ CAP exposure at 144 h (r = 0.56 and 0.55, *p* < 0.05) and a negative correlation with the control group (r = −0.54, *p* < 0.05). At 48 h of CAP exposure, the brown module was significantly activated at LC_10_ (r = 0.65, *p* < 0.01), and the blue module at LC_50_ (r = 0.65, *p* < 0.01), respectively, indicating their dominant roles in short-term acute toxicity responses. The turquoise and other modules showed no significant associations with the traits, mainly participating in the maintenance of basic life activities (Figure 3e). These results indicated that gene expression profiles in *P. akamusi* shifted dynamically with exposure duration, revealing distinct regulatory patterns between short-term and long-term CAP stress.

To further identify the key hub genes in the co-expression network, gene annotations were extracted from the reference genome annotation database. By integrating the core module genes and their annotations, a total of 80 key hub genes were identified from three modules (Table S3). As shown in Figure 3f, tyrosine-protein phosphatase, c-Jun *N*-terminal kinase (JNK), Ras homolog family member A (RhoA), protein kinases, calcium-dependent protein kinase II (CaMKII), zinc finger proteins, and members of the rat sarcoma (Ras) family were identified as core genes of the brown module. Glycosyl hydrolase, ABC transporter, chitin synthase and trypsin were defined as hub genes in the blue module, which was associated with LC_50_ CAP stress for 48 h (Figure 3g). Hub genes within the yellow module are highly connected with protein homeostasis, mainly including the thioredoxin, proteasome activator PA28 beta subunit (PA28β), cytochrome c, protein disulfide-isomerase (PDI), and 2-oxoisovalerate dehydrogenase (Figure 3h).

### 3.4. Correlations Between DEGs and DEMs Under Prolonged CAP Exposure

**Figure 4:**
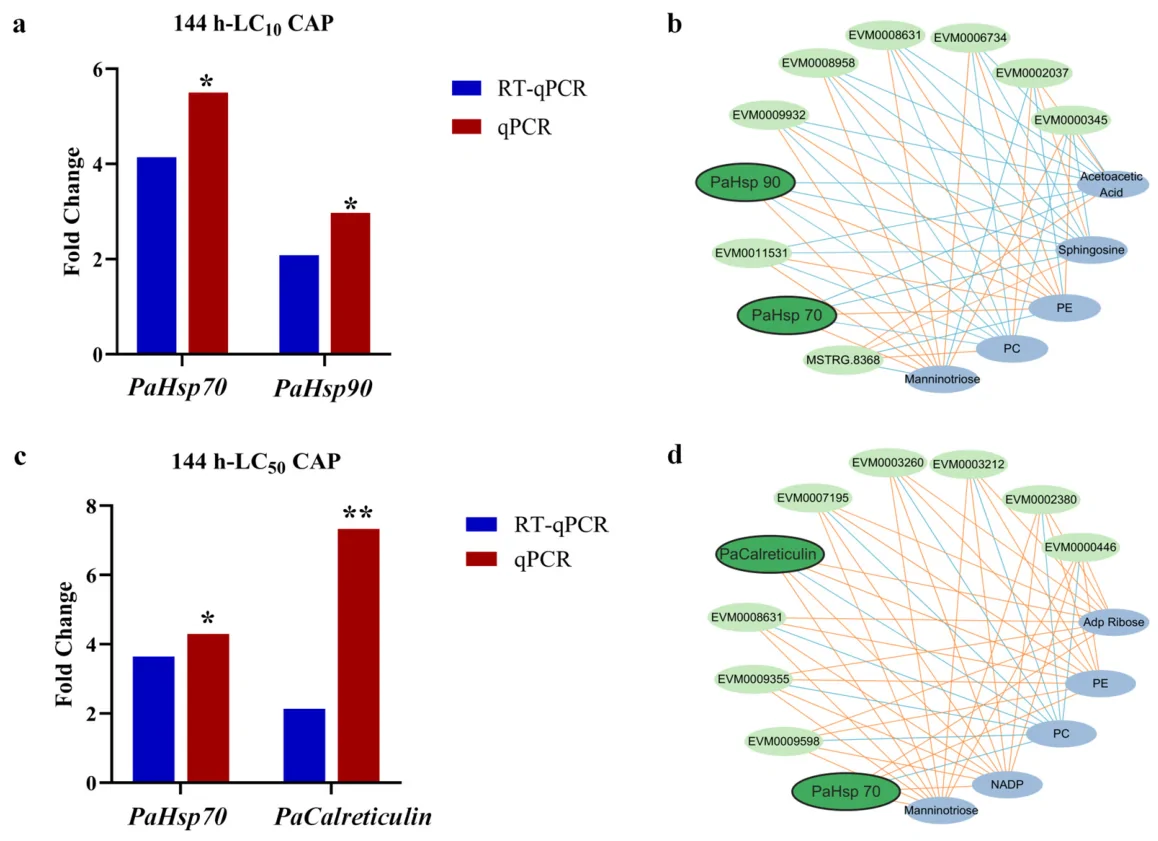
Expression validation and correlation analysis after CAP treatment for 144 h. (**a**) Expression levels of *PaHsp70* and *PaHsp90* measured by qRT-PCR and transcriptome sequencing. (**b**) Correlation analysis between DEGs and DEMs in the LC_10_ CAP-144 h treatment group. The selected metabolites include PE (18:3/16:4) and PC (16:2/16:2). (**c**) Expression levels of *PaHsp70* and PaCalreticulin measured by qRT-PCR and transcriptome sequencing. (**d**) Correlation analysis between DEGs and DEMs in the LC_50_ CAP-144 h treatment group. The selected metabolites include PE (40:6) and PC (18:3/16:4). Asterisks indicate differences between comparisons (*, *p* < 0.05, **, *p* < 0.01).

To explore the molecular response of chironomid larvae to prolonged CAP stress, we conducted integrated transcriptomic and metabolomic analyses on a series of selected DEGs and DEMs at 144 h post-exposure under LC_10_ and LC_50_ concentrations. LC_10_ CAP treatment for 144 h induced a remarkably higher expression of *PaHsp70* and *PaHsp90*, which agreed with the RT-qPCR data (Figure 4a). Correlation analysis revealed that these two genes were significantly negatively correlated with phosphatidylethanolamine (PE), acetoacetic acid, and phosphatidylcholine, indicating a metabolic reprogramming in chironomid larvae (Figure 4b and Table S4). In the 144 h LC_50_ CAP treatment group, both transcriptome and RT-qPCR revealed that *PaHsp70* as well as *calreticulin* underwent an obvious enhancement (Figure 4c). Meanwhile, they were also positively associated with alterations in ADP ribose, PE and NADP, reflecting an integrated cellular response to energy crisis and oxidative stress (Figure 4d and Table S4).

### 3.5. Functional Role of PaHsp70 in Chironomid Larvae Under CAP Stress

**Figure 5:**
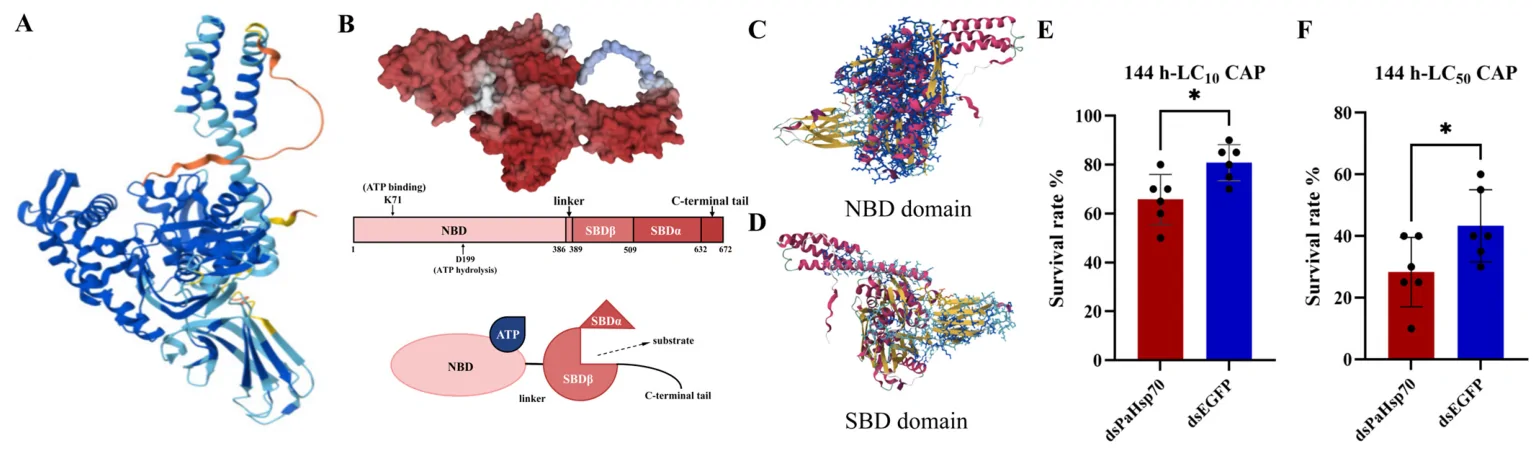
Structural features of *PaHsp70* and the effect of its silencing on larval survival under CAP stress. (**A**) 3D structure of the *PaHsp70* molecular chaperone. (**B**) Domain organization and schematic structure. (**C**) *N*-terminal nucleotide-binding domain (NBD). (**D**) C-terminal substrate-binding domain (SBD). (**E**) Survival rate of dsPaHsp70-treated larvae under LC_10_ CAP stress for 144 h. (**F**) Survival rate under LC_50_ CAP stress for 144 h. *n* = 6. Asterisks indicate differences between comparisons (*, *p* < 0.05).

The structure of *PaHsp70* was constructed according to its amino acid sequence. *PaHsp70* exhibited a characteristic bilobed modular structure composed of two functionally independent structural domains linked by a flexible interdomain region, an *N*-terminal nucleotide-binding domain (NBD) and a C-terminal substrate-binding domain (SBD) (Figure 5A–D). To further investigate the functional role of *PaHsp70* under CAP stress, we knocked down *PaHsp70* expression by RNAi. PaHsp70 expression was significantly reduced in dsPaHsp70-treated larvae compared to dsEGFP controls (Supplementary Figure S1). Following *PaHsp70* RNAi for 144 h, the survival rates of chironomid larvae exposed to LC_10_ and LC_50_ CAP significantly decreased from 80.8% to 65.8% and from 43.3% to 28.3%, respectively (Figure 5E,F).

## 4. Discussion

A central mechanism of pesticide toxicity is oxidative stress. Research has demonstrated that pesticides could induce a significant surge in ROS within non-target aquatic organisms, a threat countered by the action of endogenous antioxidant enzymes. The surge in ROS further triggers the activation of the host antioxidant defense system, as evidenced by the upregulation of enzymatic activities including superoxide dismutase (SOD), catalyse, and POD [31,32]. Nevertheless, sustained or high-concentration exposure can overwhelm this protective capacity, resulting in persistent oxidative injury and cellular apoptosis in insects [33]. CAP is a commonly used diamide insecticide in agriculture and rice–fish co-culture systems. Its environmental residues, with concentrations reaching up to microgram levels, raise ecological concerns due to their persistence and potential toxicity. The physiological results of our study showed that following sub-lethal CAP stress, the activities of POD and GSH-Px were significantly enhanced from 24 h of exposure onward. The subsequent recovery of GSH-Px to baseline at 144 h suggests that larvae might be able to restore redox homeostasis, possibly through GSH-Px resynthesis, GSH replenishment, or compensatory activation of other antioxidant enzymes. Meanwhile, protein oxidative damage, as indicated by protein carbonyl content, remained unchanged during the entire 144 h challenge period. In contrast, exposure to the LC_50_ concentration of CAP led to a marked increase in protein carbonyl content, along with activated POD and GSH-Px (Figure 1). Consistently, previous studies on chironomid larvae demonstrated that high pesticide concentration also induced a more significant enhancement of SOD and glutathione S-transferase activities than low concentration did. Lipid peroxidation was not induced by the low concentration of contaminants but was markedly elevated in the high-concentration group [17,21]. Taken together with our POD, GSH-Px, and protein carbonyl data presented here, these earlier findings indicate that CAP could induce a consistent concentration-dependent oxidative stress profile in chironomid larvae, despite differences in the specific biomarkers measured. In addition, the glutathione metabolism pathway was remarkably enriched after 48 h exposure to LC_10_ CAP, indicating a vital role of antioxidant enzymes in mitigating CAP toxicity at an early stage. These results suggest that the antioxidant capacity was sufficient to counteract the toxic effects of oxidative stress under LC_10_ conditions for a relatively chronic course, but was overwhelmed at the LC_50_ concentration.

Previous studies have demonstrated that the JNK signaling pathway contributes to antioxidant defense by glutathione maintenance and ROS reduction [34,35]. In *Nilaparvata lugens*, synergistic regulation of Cytochrome P450 (P450) gene expression by JNK and extracellular signal-regulated kinase (ERK) has been shown to confer resistance to the insecticide nitenpyram [36]. Mitogen-activated protein kinase (MAPK) pathways are involved in neonicotinoid resistance via activating P450 genes in the whitefly *Bemisia tabaci* [37]. In the present study, genes upregulated following LC_10_ CAP treatment were significantly enriched in drug metabolism and glutathione metabolism pathways. Notably, several protein kinases, including JNK, and RhoA were identified as core genes within the module that had a strong correlation with LC_10_ CAP treatment. Accordingly, we propose that a sublethal dosage of CAP might induce phosphorylation of JNK and then activate downstream signaling cascades in chironomid larvae, thereby promoting the expression of genes involved in antioxidant defense and detoxification processes. Previous studies in *Drosophila* have demonstrated that Rho GTPases, and their crucial member RhoA, can regulate the JNK signaling pathway, and that JNK activation subsequently induces cell apoptosis [38,39]. In line with these findings, our results showed that sublethal CAP stress at the initial stage induced significant alteration of genes involved in lysosome function. Based on these correlative observations, we hypothesize that RhoA may activate the JNK signaling cascade, ultimately leading to apoptosis. The upregulation of lysosomal genes might be associated with either the execution of apoptosis or the cellular clearance of apoptotic cells. Collectively, these observations suggest that chironomid larvae might employ this signaling pathway in response to short-term exposure to sublethal doses of CAP. Direct functional and histological evidence is needed to validate this proposed pathway in future studies.

Metabolic pathway analysis revealed that glycolysis and pyruvate metabolism were significantly enriched in chironomid larvae following 48 h of exposure to the LC_50_ concentration of CAP. This enrichment suggests an elevated demand for ATP production through carbohydrate catabolism under LC_50_ CAP stress. Notably, the glycosyl hydrolase gene, identified as a hub gene in the co-expression network, did not exhibit a statistically significant change at the transcriptional level. Given its central network position and the concurrent activation of glycolytic pathways, the glycosyl hydrolase may act as a functional anchor of carbohydrate catabolism, enabling the host to meet acute energy demands under stress conditions. It has been generally recognized that exposure to LC_50_ levels of insecticides can trigger extensive metabolic reprogramming in aquatic insects. In the present study, following 48 h of LC_50_ CAP challenge, pathway enrichment analysis revealed that biosynthesis of cofactors, fatty acid biosynthesis, and tryptophan metabolism were remarkably enriched in chironomid larvae. These three metabolic pathways play crucial roles in maintaining cellular homeostasis and stress adaptation. For instance, fatty acids serve as key energy substrates and signaling molecules that influence systemic metabolism [40]. Additionally, we identified three trypsin genes as core responsive genes, suggesting that protein digestion and proteolytic metabolism are also highly mobilized during stress adaptation [41]. Taken together, 48 h of LC_50_ CAP treatment induced broad metabolic reprogramming and caused negative effects on the host.

Excess ROS generated by CAP can oxidatively damage proteins and promote misfolding. In response, proteasome-mediated protein degradation serves as an acute cellular mechanism to counteract such protein damage or misfolding under oxidative stress [42]. Studies on zebrafish have revealed an increase in proteasome components, accompanied by a compensatory upregulation of proteolytic capacity [43]. Beyond vertebrates, proteasome subunits also play essential roles in insect development and homeostasis. For instance, aged flies exhibited 26S proteasome disassembly, which impaired their ability to cope with proteotoxic damage induced by chronic environmental stress [44]. Reduced expression of a proteasome 20S subunit gene in the 28-spotted potato ladybird was associated with feeding impairments, stunted growth, and disrupted intestinal development [45]. The activity of the 20S proteasome depends on activating factors, among which proteasome activator subunit 28 (PA28) is a vital component. Previous research has indicated that alterations in PA28 may induce oxidative protein aggregation in the brain of a diabetic rat model [46]. The absence of PA28 in *Plasmodium falciparum* due to gene knockout increased the sensitivity of parasites to the antimalarial agent dihydroartemisinin, which additionally indicated a protective role of PA28 against proteotoxic stress [47]. Ubiquitin serves as a classical molecular tag for protein degradation. Misfolded or unfolded proteins, once ubiquitinated, are targeted for degradation via either the proteasome or the lysosome [48]. In the present study, a series of DEGs following LC_10_ CAP subchronic exposure were enriched in proteasome and ribosome pathways, suggesting enhanced clearance of damaged proteins and de novo protein synthesis. Moreover, WGCNA indicated that in the most significantly associated module, PA28β was identified as a hub gene and other genes with notable expression changes were enriched in the ubiquitin-mediated proteolysis, peroxisome and lysosome pathways. Numerous studies have investigated how the insect energy cycle responds to various stressors, including temperature extremes, UV radiation, polystyrene, pathogen infection, and others [49,50,51,52]. In the present study, following subchronic exposure to CAP, the oxidative phosphorylation pathway was significantly enriched in chironomid larvae, indicating an increased energy demand. Our data suggest that the elevated oxidative phosphorylation under long-term LC_10_ CAP exposure provides the necessary energetic support for proteasomal and lysosomal activities, thereby facilitating the elimination of damaged proteins and cellular debris.

HSPs function as essential molecular chaperones, safeguarding protein integrity in insects exposed to diverse stressors. Their primary roles include promoting the proper folding of nascent polypeptides, preventing the aggregation of denatured proteins under stress conditions, and facilitating cellular recovery after stress exposure [53]. Previous studies have reported that Hsp70 plays essential roles in pest mites and soybean aphids when dealing with heat and chemical stressors [54,55]. In this study, we discovered that in chironomid larvae exposed to subchronic CAP, *PaHsp70* expression was substantially upregulated, and its downregulation significantly reduced larval survival rate, indicating that *PaHsp70* is crucial for tolerance to this chemical stressor. Furthermore, we found that *PaHsp70* levels were significantly associated with pyruvate, phosphatidylcholine, and phosphatidylethanolamine. Given that pyruvate is a key glycolytic intermediate and phospholipids are major membrane constituents, these associations suggest that *PaHsp70* may orchestrate a metabolic shift upon CAP exposure. Similar to findings in mulberry pyralids, where Hsp70 represses anabolic pathways to prioritize protein repair [56], our results imply a redirection of resources from lipid and carbohydrate metabolism toward maintaining protein homeostasis. Based on these results, we propose that chironomid larvae adopt an adaptive strategy under sublethal CAP stress. The antioxidant system is rapidly activated to counter CAP-induced ROS. Under subchronic CAP exposure, ubiquitinated damaged proteins are either degraded by the proteasome or refolded by HSPs, while de novo protein synthesis ensures timely replenishment of the cellular protein pool.

Transcriptomic data revealed significant alterations in the TCA cycle metabolism pathway in chironomid larvae exposed to the LC_50_ concentration of CAP. This finding was corroborated by the increased accumulation of NADH, citric acid, cis-aconitic acid, and malic acid [17]. The perturbation of the TCA cycle suggests that substantial energy is required to support protein processing in the ER, ribosome-mediated protein synthesis, and other pathways involved in CAP defense. However, given the close interconnections between the TCA cycle and glycolysis, amino acid metabolism, and lipid metabolism, the observed enhancement of the TCA cycle implies a state of metabolic imbalance. Furthermore, this enhanced metabolic flux may subsequently elevate ROS levels, thereby exacerbating oxidative stress and cellular damage in chironomid larvae. Similar phenomena have been reported in studies on aquatic organisms, such as planktonic diatoms and the dinoflagellate *Alexandrium catenella* [57,58]. Notably, under prolonged LC_50_ CAP exposure, the ER stress marker *PaCalreticulin* was significantly upregulated and showed a positive correlation with ADP, NADP and manniotriose. We therefore infer that while the activation of oxidative phosphorylation and the TCA cycle represents an adaptive response to meet the increased energy demand for detoxification and cellular repair, it may also come at the cost of heightened oxidative injury and disrupted metabolism, particularly under exposure to LC_50_ concentrations of the pesticide.

However, several limitations warrant consideration. The use of only two CAP concentrations precluded dose–response modeling. In addition, although our data implicate Hsp-mediated pathways in the stress response, the complete upstream regulatory networks and downstream signaling cascades remain to be experimentally defined.

## 5. Conclusions

The effects of sublethal and lethal concentrations of CAP on chironomid larvae *Propsilocerus akamusi* exhibit distinct time- and concentration-dependent patterns. Under LC_10_ CAP exposure, the 48 h treatment primarily activates antioxidative enzymes and drug metabolism pathways, along with regulation of JNK-mediated phosphorylation signaling, as an early stress response. In contrast, the 144 h LC_10_ CAP treatment further activates the proteasome, oxidative phosphorylation, and ribosomal pathways. Upregulation of *PaHsp70* and *PaHsp90*, along with significant negative correlations with pyruvate, phosphatidylcholine, and sphingosine phosphate, indicates a metabolic remodeling that maintains protein homeostasis to support survival. This was confirmed by the increased mortality upon *PaHsp70* knockdown. Under LC_50_ CAP exposure, the 48 h treatment mainly interferes with metabolic pathways, whereas the 144 h LC_50_ CAP treatment significantly impacts protein processing in the ER and the TCA cycle. The marked upregulation of *PaCalreticulin* and *PaHsp70*, positively correlated with ADP, PE, and NADP, reflects a severe energy crisis coupled with ER stress. In summary, the toxicity of CAP to chironomid larvae intensified with prolonged exposure, and the underlying stress responses and survival strategies differed fundamentally between concentrations. Metabolic remodeling and proteostasis supported survival under prolonged sub-lethal exposure, whereas prolonged semi-lethal exposure led to a vicious cycle of energy failure and ER dysfunction.

## Figures and Tables

**Table 1:** Primers used in this study.

Primer Name	Sequence (5′–3′)	Tm
EVM0011063-F (PaHsp90-F)	AAGACGAGCAGCAGGAGAAT	60
EVM0011063-R (PaHsp90-R)	CAATGGCTCAAAACGACTCT	60
EVM0011825-F (PaHsp70-F)	ACCATATACAGACTGATAAC	58
EVM0011825-R (PaHsp70-R)	GAGCATGGAATGATCGTCTG	58
EVM0007624-F (PaCalreticulin-F)	ATTTTGGGTGCTGTCGCC	60
EVM0007624-R (PaCalreticulin-R)	CGCCGCAATCAATGTTCTGT	60
Paβ-actin-F	TCTTCCAGCCATCCTTCTTG	60
Paβ-actin-R	CGGTGATTTCCTTCTGCATT	60
dsPaHsp70-F	TAATACGACTCACTATAGGGAGGATAAAGACAAAGATAAG	-
dsPaHsp70-R	TAATACGACTCACTATAGGGCACGATTTCCTGTTCCCTTG	-

**Table 2:** The number of DEGs in *P. akamusi* chironomid larvae treated with CAP.

Group	Total Detected Genes	Total Number of DEGs	Up Regulated DEGs	Down Regulated DEGs
48 h LC_10_ CAP vs. Control	11,942	54	26	28
48 h LC_50_ CAP vs. Control	11,942	289	146	143
144 h LC_10_ CAP vs. Control	11,942	699	193	506
144 h LC_50_ CAP vs. Control	11,942	539	190	349

## Data Availability

The raw transcriptome sequence reads could be archived in the NCBI SRA database under the accession numbers PRJNA1460572 and PRJNA1348783. The metabolome raw data have been deposited in the Metabolomics Workbench repository under the study ID ST004511.

## Supplementary Materials

The following supporting information can be downloaded at https://www.mdpi.com/article/10.3390/ani16172779/s1, Figure S1: RNAi-mediated gene silencing efficiency of PaHsp70 at 12, 24 and 48 h. Table S1: The toxicology of CAP on *Propsilocerus akamusi* larvae. Table S2: Sequencing quality metrics of transcriptome data for *P. akamusi* larvae under CAP exposure; Table S3: Representative hub genes in the blue, brown, and yellow modules identified by WGCNA analysis. Table S4: Gene symbols and functional annotations of genes involved in gene-metabolite association networks under different CAP treatments.

## Abbreviations

The following abbreviations are used in this manuscript:

CAP	chlorantraniliprole
Hsp	Heat shock protein
WGCNA	Weighted Gene Co-expression Network Analysis
DEGs	Differentially expressed genes
PE	Phosphatidylethanolamine
PC	Phosphatidylcholine
