# Supplementary material for: Integrated Time-Series Biochemical, Transcriptomic and Metabolomic Analyses Reveal Key Pathways and Concentration-Dependent Transitions in Chironomid Larvae (Propsilocerus akamusi) Under Chlorantraniliprole Stress
Source: Animals (Basel). 2026 Sep 3;16(17):2779. doi: 10.3390/ani16172779 (PMC13565220; doi:10.3390/ani16172779)
Supplement: Supplementary file 1 [file animals-16-02779-s001.zip › Figure S1 and Table S2-S4.pdf]

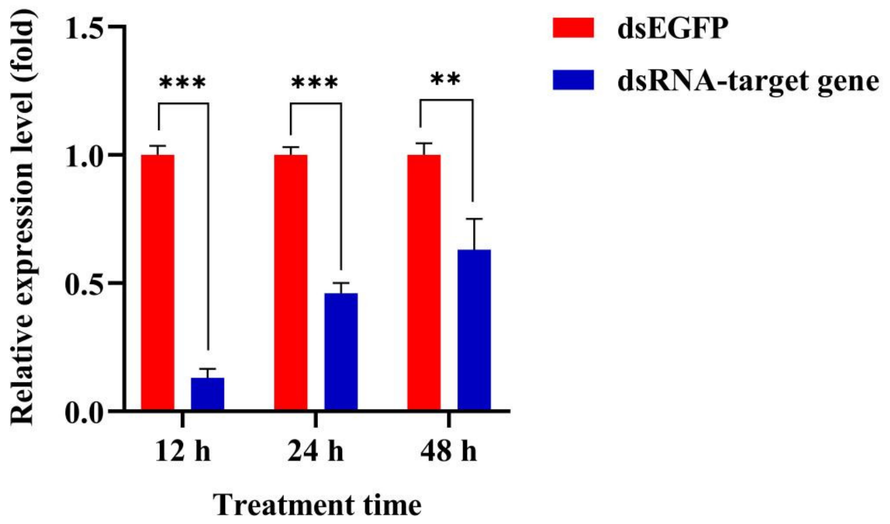

Supplementary Figure S1 RNAi-mediated gene silencing efficiency of PaHsp70 at 12, 24 and 48 h

Supplementary Table S2 Sequencing quality metrics of transcriptome data for *P. akamusi* larvae under CAP exposure

Treatment group	Clean reads	Q30 (%)	Total mapped reads (%)
Control-1	41,945,826	96.63	97.16
Control-2	41,829,412	96.53	97.09
Control-3	39,149,490	96.44	96.96
48 h LC_{10} CAP-1	41047114	96.63	97.21
48 h LC_{10} CAP-2	42020966	96.58	97.15
48 h LC_{10} CAP-3	44124078	96.56	97.01
48 h LC_{50} CAP-1	42565858	96.61	97.04
48 h LC_{50} CAP-2	39327856	96.62	97.22
48 h LC_{50} CAP-3	41453742	96.61	97.05
144 h LC_{10} CAP-1	43,468,142	96.53	96.86
144 h LC_{10} CAP-2	40,229,204	96.34	97.14
144 h LC_{10} CAP-3	43,724,280	96.55	96.94
144 h LC_{50} CAP-1	43,217,144	96.64	97.36
144 h LC_{50} CAP-2	40,102,986	96.54	97.25
144 h LC_{50} CAP-3	43,237,356	96.58	97.29

Supplementary Table S3 Representative hub genes in the blue, brown, and yellow modules identified by WGCNA analysis

Number	Gene ID	Module	Pfam_annotation	KEGG_pathway_annotation
1	EVM0005479	brown	JNK	MAPK signaling pathway - fly (ko04013); FoxO signaling pathway (ko04068); Mitophagy - animal (ko04137); Autophagy - animal (ko04140); Protein processing in endoplasmic reticulum (ko04141); Apoptosis - fly (ko04214); Apoptosis - multiple species (ko04215); Wnt signaling pathway (ko04310); Hippo signaling pathway - fly (ko04391); Toll and Imd signaling pathway (ko04624)
2	EVM0004864	brown	RING-variant domain	Ubiquitin mediated proteolysis (ko04120)
3	EVM0009403	brown	Isochorismatase family	RNA degradation (ko03018)
4	EVM0001441	brown	Tyrosine-protein phosphatase	MAPK signaling pathway - fly (ko04013); FoxO signaling pathway (ko04068); Insulin signaling pathway (ko04910); Apoptosis - fly (ko04214)
5	EVM0004773	brown	Fes/CIP4, and EFC/F-BAR homology domain	Endocytosis (ko04144); Regulation of actin cytoskeleton (ko04810); Fc gamma R-mediated phagocytosis (ko04666)
6	EVM0010001	brown	protein kinase	MAPK signaling pathway - fly (ko04013); TGF-beta signaling pathway (ko04350)
7	EVM0000111	brown	AF-4 proto-oncoprotein C-terminal region	Transcriptional misregulation in cancer (ko05202); Hematopoietic cell lineage (ko04640); Notch signaling pathway (ko04330)
8	EVM0003523	brown	N-terminal C2 in EEIG1 and EHBP1 proteins	Endocytosis (ko04144); Intracellular trafficking, secretion, and vesicular transport (ko04130); Phagosome (ko04145)
9	EVM0008428	brown	RNSP1-SAP18 binding (RSB) motif	RNA transport (ko03013); mRNA surveillance pathway (ko03015); Spliceosome (ko03040)
10	EVM0000308	brown	Nebulin repeat	Regulation of actin cytoskeleton (ko04810); Cardiac muscle contraction (ko04260); Vascular smooth muscle contraction (ko04270)
11	EVM0004782	brown	TLD	ECM-receptor interaction (ko04512); Focal adhesion (ko04510); TGF-beta signaling pathway (ko04350); Wnt signaling pathway (ko04310)
12	EVM0008359	brown	Tyrosine-protein phosphatase	MAPK signaling pathway - fly (ko04013); FoxO signaling pathway (ko04068); Insulin signaling pathway (ko04910); Apoptosis - fly (ko04214)
13	EVM0007691	brown	Acetyl-CoA hydrolase/transferase C-terminal domain	Fatty acid metabolism (ko00061); Citrate cycle (TCA cycle) (ko00020); Pyruvate metabolism (ko00620); Peroxisome (ko04146)
14	EVM0006308	brown	Saposin-like type B, region 2	Sphingolipid metabolism (ko00600); Lysosome (ko04142)
15	EVM0011449	brown	WD domain, G-beta repeat	Wnt signaling pathway (ko04310); Notch signaling pathway (ko04330); MAPK signaling pathway - fly (ko04013); Ubiquitin mediated proteolysis (ko04120)
16	EVM0002567	brown	Protein kinase	Wnt signaling pathway (ko04310); Phototransduction - fly (ko04745)

17	EVM0009038	brown	Neuroendocrine protein 7B2 precursor (Secretogranin V)	Neuroactive ligand-receptor interaction (ko04080); Synaptic vesicle cycle (ko04721); Insulin secretion (ko04911); Glucagon signaling pathway (ko04922)
18	EVM0002570	brown	CaMKII	FoxO signaling pathway (ko04068); Endocytosis (ko04144); TGF-beta signaling pathway (ko04350)
19	EVM0008517	brown	Unkempt Zinc finger domain 1 (Znf1)	Transcriptional misregulation in cancer (ko05202); Notch signaling pathway (ko04330); Wnt signaling pathway (ko04310); Hedgehog signaling pathway (ko04340)
20	EVM0008328	brown	BAH domain	Chromatin remodeling (ko03036); Transcriptional misregulation in cancer (ko05202); Notch signaling pathway (ko04330); Wnt signaling pathway (ko04310)
21	EVM0002843	brown	Piwi domain	RNA interference (ko03019); Transcriptional silencing by small RNAs (ko03020); PI3K-Akt signaling pathway (ko04151); Apoptosis - fly (ko04214)
22	EVM0008255	brown	NACHT domain	NOD-like receptor signaling pathway (ko04621); Toll and Imd signaling pathway (ko04624); Inflammatory mediator regulation of TRP channels (ko04750); Apoptosis - fly (ko04214)
23	EVM0009792	brown	Phosphatidylinositol 3- and 4-kinase	Inositol phosphate metabolism (ko00562); Phosphatidylinositol signaling system (ko04070)
24	EVM0007181	brown	RhoGAP	Regulation of actin cytoskeleton (ko04810); Focal adhesion (ko04510); Endocytosis (ko04144); MAPK signaling pathway - fly (ko04013)
25	EVM0002168	brown	Zinc finger	Transcriptional misregulation in cancer (ko05202); Notch signaling pathway (ko04330); Wnt signaling pathway (ko04310); Hedgehog signaling pathway (ko04340)
26	EVM0007442	brown	RhoA	Endocytosis (ko04144); mTOR signaling pathway (ko04150); Wnt signaling pathway (ko04310); TGF-beta signaling pathway (ko04350)
27	EVM0011887	blue	PAS domain	Circadian rhythm - fly (ko04711)
28	EVM0011805	blue	Pentapeptide repeats (9 copies)	Cell adhesion molecules (CAMs) (ko04514); ECM-receptor interaction (ko04512); Focal adhesion (ko04510); Leukocyte transendothelial migration (ko04670)
29	EVM0005613	blue	Trypsin	Protein digestion and absorption (ko04974); ECM-receptor interaction (ko04512); Focal adhesion (ko04510); Complement and coagulation cascades (ko04610); Antigen processing and presentation (ko04612)
30	EVM0005972	blue	Transcription activator MBF2	Transcriptional misregulation in cancer (ko05202); Notch signaling pathway (ko04330); Wnt signaling pathway (ko04310); Hedgehog signaling pathway (ko04340)
31	EVM0000071	blue	UAA transporter family	Amino acid metabolism (ko00260); ABC transporters (ko02010); Intestinal immune network

				for IgA production (ko04672); Protein digestion and absorption (ko04974)
32	EVM0008344	blue	Glutamine amidotransferases class-II	Alanine, aspartate and glutamate metabolism (ko00250); Nitrogen metabolism (ko00910); Biosynthesis of amino acids (ko01230)
33	EVM0008846	blue	Zinc carboxypeptidase	Protein digestion and absorption (ko04974); ECM-receptor interaction (ko04512); Focal adhesion (ko04510); Complement and coagulation cascades (ko04610)
34	EVM0009773	blue	N-acetylmuramoyl-L-alanine amidase	Toll and Imd signaling pathway (ko04624)
35	EVM0006076	blue	Chitin synthase	Amino sugar and nucleotide sugar metabolism (ko00520)
36	EVM0004129	blue	Methuselah N-terminus	G protein-coupled receptor signaling pathway (ko04010); MAPK signaling pathway - fly (ko04013); FoxO signaling pathway (ko04068); Toll and Imd signaling pathway (ko04624); Longevity regulating pathway - fly (ko04212)
37	EVM0000315	blue	Trypsin	Neuroactive ligand-receptor interaction (ko04080)
38	EVM0008861	blue	SAM domain (Sterile alpha motif)	MAPK signaling pathway - fly (ko04013); Wnt signaling pathway (ko04310); Notch signaling pathway (ko04330); Transcriptional misregulation in cancer (ko05202)
39	EVM0000305	blue	BTB/POZ domain	Transcriptional misregulation in cancer (ko05202); Notch signaling pathway (ko04330); Wnt signaling pathway (ko04310); Cell cycle (ko04110)
40	EVM0010272	blue	ABC transporter	ABC transporters (ko02010)
41	EVM0008065	blue	SMP-30/Gluconolactonase/LRE-like region	Pentose phosphate pathway (ko00030); Ascorbate and aldarate metabolism (ko00053); Carbon metabolism (ko01200)
42	EVM0002485	blue	Glycosyl hydrolases	Pentose and glucuronate interconversions (ko00040); Glycosaminoglycan degradation (ko00531); Porphyrin and chlorophyll metabolism (ko00860); Drug metabolism - other enzymes (ko00983); Lysosome (ko04142)
43	EVM0005680	blue	Trypsin	Neuroactive ligand-receptor interaction (ko04080)
44	EVM0005511	blue	Histidine phosphatase superfamily (branch 2)	Riboflavin metabolism (ko00740); Lysosome (ko04142)
45	EVM0011583	blue	Astacin (Peptidase family M12A)	ECM-receptor interaction (ko04512); Focal adhesion (ko04510); Complement and coagulation cascades (ko04610); Antigen processing and presentation (ko04612); Wnt signaling pathway (ko04310)
46	EVM0010134	blue	Insect allergen related repeat, nitrile-specifier detoxification	Xenobiotics biodegradation and metabolism (ko00980); Drug metabolism - cytochrome P450 (ko00982); Retinol metabolism (ko00830); Antigen processing and presentation (ko04612); Allograft rejection (ko05330)
47	EVM0007599	blue	Peptidase family M28	Protein digestion and absorption (ko04974);

				ECM-receptor interaction (ko04512); Focal adhesion (ko04510); Complement and coagulation cascades (ko04610)
48	EVM0003498	blue	Chitin binding Peritrophin	Chitin metabolism (ko00520); Amino sugar and nucleotide sugar metabolism (ko00520); ECM-receptor interaction (ko04512); Focal adhesion (ko04510); Toll and Imd signaling pathway (ko04624)
49	EVM0000472	blue	Chitin binding Peritrophin	Chitin metabolism (ko00520); Amino sugar and nucleotide sugar metabolism (ko00520); ECM-receptor interaction (ko04512); Focal adhesion (ko04510); Toll and Imd signaling pathway (ko04624)
50	EVM0008963	blue	Dipeptidyl peptidase IV (DPP IV) N-terminal region	Protein digestion and absorption (ko04974); Cell adhesion molecules (CAMs) (ko04514); Focal adhesion (ko04510); Antigen processing and presentation (ko04612); Complement and coagulation cascades (ko04610)
51	EVM0001827	blue	Tubulin/FtsZ family, GTPase domain	Regulation of actin cytoskeleton (ko04810); Cell cycle (ko04110); Intracellular trafficking, secretion, and vesicular transport (ko04130); Endocytosis (ko04144); Phagosome (ko04145)
52	EVM0000642	yellow	Carboxypeptidase regulatory-like domain	Protein digestion and absorption (ko04974); ECM-receptor interaction (ko04512); Focal adhesion (ko04510); Complement and coagulation cascades (ko04610)
53	EVM0001054	yellow	Protein kinase domain	Ribosome biogenesis in eukaryotes (ko03008); Mitophagy - animal (ko04137); Wnt signaling pathway (ko04310)
54	EVM0001113	yellow	EF-1 guanine nucleotide exchange domain	Ribosome (ko03010); Protein processing in endoplasmic reticulum (ko04141); RNA transport (ko03013); mRNA surveillance pathway (ko03015)
55	EVM0001377	yellow	PX domain	Endocytosis (ko04144); Intracellular trafficking, secretion, and vesicular transport (ko04130); Phagosome (ko04145); MAPK signaling pathway - fly (ko04013); PI3K-Akt signaling pathway (ko04151)
56	EVM0001764	yellow	Elongation factor 1 gamma, conserved domain	Ribosome (ko03010); Protein processing in endoplasmic reticulum (ko04141); RNA transport (ko03013); mRNA surveillance pathway (ko03015)
57	EVM0002142	yellow	C-terminal region of MMR_HSR1 domain	Ribosome (ko03010); RNA transport (ko03013); mRNA surveillance pathway (ko03015); Nucleotide excision repair (ko03420)
58	EVM0002292	yellow	Leucine Rich repeats (2 copies)	Aminoacyl-tRNA biosynthesis (ko00970)
59	EVM0002718	yellow	Domain of unknown function (DUF5600)	Endocytosis (ko04144)
60	EVM0003848	yellow	Mitochondrial 28S ribosomal protein S27	Ribosome (ko03010); Oxidative phosphorylation (ko00190); Citrate cycle (TCA cycle) (ko00020); Fatty acid metabolism (ko00061)

61	EVM0003852	yellow	Thioredoxin	Protein processing in endoplasmic reticulum (ko04141)
62	EVM0004635	yellow	SNARE domain	SNARE interactions in vesicular transport (ko04130); Phagosome (ko04145)
63	EVM0004776	yellow	Cytochrome c	Apoptosis - fly (ko04214); Apoptosis - multiple species (ko04215)
64	EVM0005257	yellow	WD domain, G-beta repeat	RNA degradation (ko03018)
65	EVM0005611	yellow	2-oxoacid dehydrogenases	Valine, leucine and isoleucine degradation (ko00280); Propanoate metabolism (ko00640)
66	EVM0006277	yellow	Metallopeptidase family M24	Protein digestion and absorption (ko04974); ECM-receptor interaction (ko04512); Focal adhesion (ko04510); Complement and coagulation cascades (ko04610)
67	EVM0006688	yellow	CD36 family	Phagosome (ko04145)
68	EVM0007462	yellow	EF-hand domain pair	Calcium signaling pathway (ko04020); MAPK signaling pathway - fly (ko04013); Regulation of actin cytoskeleton (ko04810); Cardiac muscle contraction (ko04260); Vascular smooth muscle contraction (ko04270)
69	EVM0007499	yellow	Thioredoxin	Protein processing in endoplasmic reticulum (ko04141)
70	EVM0008708	yellow	Translocon-associated protein (TRAP), alpha subunit	Protein processing in endoplasmic reticulum (ko04141)
71	EVM0008865	yellow	Gtr1/RagA G protein conserved region	Autophagy - animal (ko04140); mTOR signaling pathway (ko04150)
72	EVM0008876	yellow	Repeat in HS1/Cortactin	Regulation of actin cytoskeleton (ko04810); Focal adhesion (ko04510); Endocytosis (ko04144); Phagosome (ko04145); MAPK signaling pathway - fly (ko04013)
73	EVM0008981	yellow	FKBP-type peptidyl-prolyl cis-trans isomerase	Protein processing in endoplasmic reticulum (ko04141); Antigen processing and presentation (ko04612); T cell receptor signaling pathway (ko04660); B cell receptor signaling pathway (ko04662); MAPK signaling pathway - fly (ko04013)
74	EVM0009347	yellow	CAAX prenyl protease N-terminal, five membrane helices	Terpenoid backbone biosynthesis (ko00900)
75	EVM0009467	yellow	Proteasome activator pa28 beta subunit	Proteasome (ko03050)
76	EVM0009491	yellow	Dehydrogenase E1 component	Valine, leucine and isoleucine degradation (ko00280); Propanoate metabolism (ko00640)
77	EVM0009923	yellow	KIF-1 binding protein C terminal	Intracellular trafficking, secretion, and vesicular transport (ko04130); Endocytosis (ko04144); Phagosome (ko04145); Regulation of actin cytoskeleton (ko04810); Cell cycle (ko04110)
78	EVM0009948	yellow	Nanos RNA binding domain	RNA transport (ko03013); mRNA surveillance pathway (ko03015); Translational control (ko03017); Notch signaling pathway (ko04330);

79	EVM0011343	yellow	eIF4-gamma/eIF5/eIF2-epsilon	Wnt signaling pathway (ko04310) RNA transport (ko03013); Herpes simplex virus 1 infection (ko05168)
80	EVM0011644	yellow	Lens epithelium-derived growth factor (LEDGF)	MAPK signaling pathway - fly (ko04013); PI3K-Akt signaling pathway (ko04151); Apoptosis - fly (ko04214); Transcriptional misregulation in cancer (ko05202); Wnt signaling pathway (ko04310)

Supplementary Table S4 Gene symbols and functional annotations of genes involved in gene-metabolite association networks under different CAP treatments

Gene ID	Treatment group	Pfam_annotation
EVM0003260	LC_{10}CAP-144 h	Tetraspanin family
EVM0003212	LC_{10}CAP-144 h	Leucine rich repeat
EVM0007195	LC_{10}CAP-144 h	Dual specificity phosphatase, catalytic domain
EVM0002380	LC_{10}CAP-144 h	JAKMIP CC3 domain
EVM0000446	LC_{10}CAP-144 h	Protein tyrosine and serine/threonine kinase
EVM0008631	LC_{10}CAP-144 h	Chaperone for wingless signalling and trafficking of LDL receptor
EVM0009355	LC_{10}CAP-144 h	Protein tyrosine and serine/threonine kinase
EVM0009598	LC_{10}CAP-144 h	Laminin EGF domain
EVM0008631	LC_{50}CAP-144 h	Chaperone for wingless signalling and trafficking of LDL receptor
EVM0006734	LC_{50}CAP-144 h	Cytochrome c
EVM0008958	LC_{50}CAP-144 h	Trypsin
EVM0002037	LC_{50}CAP-144 h	peroxisome
EVM0009932	LC_{50}CAP-144 h	RPEL repeat
EVM0000345	LC_{50}CAP-144 h	Protein kinase domain
EVM0011531	LC_{50}CAP-144 h	Endoplasmic Reticulum Oxidoreductin 1 (ERO1)
MSTRG.8368	LC_{50}CAP-144 h	Novel transcribed sequence, putative long non-coding RNA
