# Supplementary material for: Integrated Time-Series Biochemical, Transcriptomic and Metabolomic Analyses Reveal Key Pathways and Concentration-Dependent Transitions in Chironomid Larvae (Propsilocerus akamusi) Under Chlorantraniliprole Stress
Source: Animals (Basel). 2026 Sep 3;16(17):2779. doi: 10.3390/ani16172779 (PMC13565220; doi:10.3390/ani16172779)
Supplement: Supplementary file 1 [file animals-16-02779-s001.zip › Table S1.pdf]

Supplementary Table S1 The toxicology of CAP on *Propsilocerus akamusi* larvae

Concentration (μg/L)	0	5	20	40	100	125	500	1000	2000	Regression equation	LC_{50} (μg/L)	95% confidence interval	
24 h	0	0	1	1	1	1	3	4	15				
	0	0	0	1	2	2	5	7	15				
	0	0	0	0	1	3	4	10	15	y=-4.012+1.153x	3012.346	1612.067	9574.992
Cumulative mean mortality	0.00	0.00	0.33	0.67	1.33	2.00	4.00	7.00	15.00				
48 h	0	0	0	0	1	1	2	1	1				
	0	0	1	1	1	2	2	3	1				
	0	0	0	1	1	2	1	0	0	y=-3.368+0.983x	2676.026	1370.357	8863.002
Cumulative mean mortality	0.00	0.00	0.67	1.33	2.33	3.67	5.67	8.33	15.67				
72 h	0	1	1	1	2	2	1	3	3				
	0	0	1	3	2	3	2	2	2				
	0	0	0	2	2	2	2	2	2	y=-2.731+0.839x	1792.922	919.143	5556.295
Cumulative mean mortality	0.00	0.33	1.33	3.33	4.33	6.00	7.33	10.67	18.00				
96 h	1	1	3	3	4	3	3	3	3				
	0	2	3	4	3	3	4	4	5				
	1	2	2	3	4	2	4	4	3	y=-1.992+0.687x	795.539	415.599	2208.058
Cumulative mean mortality	0.67	2.00	4.00	6.67	8.00	8.67	11.00	14.33	21.67				
120 h	0	1	1	1	3	4	4	6	4				
	0	1	1	4	3	3	5	5	1				
	0	2	2	2	4	5	4	5	3	y=-1.818+0.756x	254.602	151.210	474.801
Cumulative mean mortality	0.67	3.33	5.33	9.00	11.33	12.67	15.33	19.67	24.33				
144 h	1	1	2	6	5	8	8	5	6				
	0	0	1	6	5	6	6	6	4				
	0	1	3	8	5	6	8	4	6	y=-1.795+0.988x	65.404	40.897	99.217
Cumulative mean mortality	1.00	4.00	7.33	15.67	16.33	19.33	22.67	24.67	29.67				
